# De novo urothelial carcinoma in kidney transplant recipients: a single-center retrospective cohort study

**DOI:** 10.1007/s11255-026-05026-2

**Published:** 2026-01-21

**Authors:** Jacob Schmidt, Malte Lehnert, Isabel Lichy, Irena Goranova, Sarah Weinberger, Jonathan Jeutner, Lukas Kurz, Henning Plage, Thorsten Schlomm, Frank Friedersdorff, Robert Peters, Nadine Biernath, Bernhard Ralla

**Affiliations:** 1 Department of Urology, Charité – Universitaetsmedizin Berlin, Freie Universitaet Berlin, Humboldt-Universitaet Zu Berlin, Berlin Institute of Health, Augustenburger Platz 1, 13353 Berlin, Germany; 2 Department of Urology, Evangelisches Krankenhaus Königin Elisabeth Herzberge, Berlin, Germany

**Keywords:** Kidney transplantation, Urothelial carcinoma, Upper-tract urothelial carcinoma, Kidney transplant recipients, Cancer incidence, Post-transplant malignancy

## Abstract

**Purpose:**

To characterize the incidence, clinicopathologic features, treatment patterns, and both oncologic and graft-related outcomes of de novo urothelial carcinoma (UC) in kidney transplant recipients (KTRs) at a large German transplant center.

**Methods:**

Retrospective single-center cohort of KTRs (2005–2024). 19 patients with post-transplant UC were identified among 4,012 KTRs. We extracted demographics, transplant and tumor characteristics, treatments, graft outcomes, and survival. The standardized incidence ratio (SIR) was calculated using population-based incidence rates, and Kaplan–Meier estimates were used for overall survival (OS), metastasis-free survival (MFS), and graft survival (GS).

**Results:**

UC occurred in 0.47% of KTRs, yielding an SIR of 2.21 (95% confidence interval [CI] 1.33–3.44) compared with the general population. Most tumors were bladder-based (63.2%), but there was a high proportion of upper-tract urothelial carcinoma (UTUC; 31.6%), including graft-involving tumors. Radical surgery was performed in 47.4% of patients, 15.8% received systemic chemotherapy, and intra-vesical therapy was rarely used. Median OS after UC diagnosis was 55 months, with estimated 3- and 5-year OS rates of 70% and 51%, respectively, and 3- and 5-year MFS rates of 73%. Graft failure occurred in 47.4% of patients, and in 21.1% of the total cohort it was directly attributed to UC.

**Conclusion:**

Post-transplant UC in KTRs is associated with a more than twofold increased incidence compared with the general population and a disproportionate burden of upper tract and graft-involving tumors. Management must carefully balance oncologic control against preservation of transplant function in a setting where standard intra-vesical and systemic therapies are constrained by immunosuppression. These findings support risk-adapted lifelong urologic surveillance and highlight the need for dedicated treatment strategies tailored to immunosuppressed patients.

**Supplementary Information:**

The online version contains supplementary material available at 10.1007/s11255-026-05026-2.

## Introduction

Kidney transplantation (KT) remains the gold standard for treating end-stage renal disease (ESRD), offering superior survival and quality of life compared to dialysis. However, lifelong immunosuppression alters immune surveillance and significantly increases the risk of malignancies, which have become one of the leading causes of late mortality in kidney transplant recipients (KTRs) [1–3]. Among post-transplant malignancies, urothelial carcinoma (UC)—encompassing tumors of the bladder and upper urinary tract—represents a particularly relevant clinical entity due to its aggressive behavior, diagnostic complexity, and implications for graft function and patient survival.

Numerous large-scale epidemiological studies across diverse populations have consistently shown a markedly increased incidence of UC in KTRs compared to the general population [4–6]. Geographic heterogeneity is notable: in East Asian cohorts, this elevated risk has in some series shown a female predominance and a shift toward upper tract rather than bladder-dominant disease [4, 7]. These patterns likely reflect an interplay of environmental and iatrogenic exposures—such as aristolochic acid, BK polyomavirus reactivation, and long-term immunosuppression—as well as differences in screening practices [7, 8]. In several transplant cohorts, UC has ranked among the most frequent post-transplant malignancies, underscoring its clinical relevance in the long-term care of KTRs [4].

The risk profile for UC in transplant recipients extends beyond traditional urothelial risk factors such as smoking or age. Chronic immunosuppression—particularly with calcineurin inhibitors and azathioprine—may impair tumor immune surveillance and promote carcinogenesis [1]. Additional contributors include ischemia-reperfusion injury and BK polyomavirus reactivation [9–11]. Aristolochic acid nephropathy, in particular, has been strongly associated with rapid UC development after transplantation and may explain part of the geographic variation in incidence [4, 7]. A consistent finding across studies is the prolonged latency of UC development after transplantation, often exceeding 7–10 years, underscoring the need for lifelong oncologic surveillance beyond the conventional post-transplant follow-up [12, 13].

UC involving the transplant kidney itself is a rare but highly challenging clinical scenario. It is typically detected late, often at advanced stages, and associated with poor prognosis and difficult surgical management [5, 14]. When tumors arise in the graft, the dual goals of oncological control and preservation of renal function are often in direct conflict. This dilemma also extends to upper-tract tumors in the native urinary tract, where surgical approaches such as nephroureterectomy may compromise residual renal function or necessitate dialysis.

Diagnostic and therapeutic strategies in this population must navigate the delicate balance between adequate cancer control and graft preservation. Intravesical therapies such as Bacillus Calmette–Guérin (BCG), widely used in the immunocompetent population, are often avoided in transplant patients due to concerns over systemic dissemination and unpredictable immune responses [15, 16]. Similarly, systemic chemotherapy and checkpoint inhibitors, though effective in advanced UC, are associated with significant nephrotoxicity or high rates of transplant rejection, limiting their use in KTRs [12, 17].

Despite the growing body of evidence, UC in KTRs remains understudied compared to renal cell carcinoma (RCC) [18]. Most available data stem from small series, single-center cohorts, or registry analysis with limited granularity. As a result, clinical guidelines specific to transplant-associated UC are lacking, and management decisions are often extrapolated from the immunocompetent population without validation.

The present study aims to fill this gap by characterizing the incidence, clinico-pathological features, therapeutic strategies, and outcomes of UC in a well-defined cohort of kidney transplant recipients treated at a large German transplant center. Special emphasis is placed on upper-tract disease and graft involvement, as well as cancer-specific survival, metastasis-free survival, and graft function. By integrating real-world data with existing evidence, we hope to provide clinically relevant insights for improving care and surveillance of this vulnerable patient population.

## Materials and methods

### Patients

This retrospective single-center cohort study was conducted at the Department of Urology, Charité – Universitätsmedizin Berlin. All patients who received a KT at our institution between January 2005 and May 2024 were screened for the occurrence of de novo UC during follow-up. Patients with UC after KT were identified by extraction of relevant ICD-10 diagnosis codes from the institutional electronic health record system, followed by manual chart review. All UC diagnoses were histologically confirmed based on pathology reports. Clinical data were obtained from electronic health records and the institutional transplant registry. Kidney transplant recipients at our center are routinely enrolled in a structured post-transplant follow-up program with long-term monitoring (typically including at least annual follow-up visits), which reduces the likelihood of missed malignancy diagnoses, although complete capture of events occurring entirely outside our health system cannot be guaranteed in a retrospective study. Patients transplanted at our institution were eligible irrespective of whether parts of their subsequent care occurred outside our health system; if follow-up at our center ended, patients contributed person-time until the last documented contact (‘last seen alive’) and were censored thereafter. Among a total of 4,012 KTRs, 19 patients were identified with UC diagnosed after transplantation and were included in the final analysis. Patients with a history of UC prior to KT were excluded.

Demographic variables included age, sex, and body mass index (BMI) at the time of transplantation. Transplant-specific variables comprised donor age and sex, type of donation (living vs. deceased), graft location (left or right iliac fossa), operative time, and cold ischemia time (CIT). Relevant pre-transplant information included the underlying kidney disease, dialysis modality (hemodialysis, peritoneal dialysis, or pre-emptive KT), dialysis duration, and residual diuresis.

Post-transplant variables included the immunosuppressive regimen at the time of UC diagnosis (e.g., use of tacrolimus, cyclosporine, mycophenolate mofetil) and postoperative complications within 30 days of KT, graded according to the Clavien–Dindo classification. Delayed graft function (DGF) was defined as the need for dialysis within the first 7 days after transplantation. Graft function was evaluated using serum creatinine measurements at 6 months, 1 year, 3 years, and 5 years post-transplant. Graft failure (GF) was defined as the initiation of dialysis, transplant nephrectomy, or death with a non-functioning graft. Graft survival (GS) was calculated from the date of transplantation to the date of graft failure or last follow-up.

Tumor-specific variables included patient age at UC diagnosis, latency period (time from KT to UC diagnosis), tumor location (bladder, native upper urinary tract, or transplant urinary tract involvement), and clinical presentation (e.g., macrohematuria, microhematuria). Further parameters included the number of transurethral resections of bladder tumors (TURBT), histologic subtype, tumor grade (WHO 1973 and 2016), clinical and pathological T stage, lymph node status, and the presence of distant metastases. Treatment strategies were categorized into local therapies (e.g., TURBT, intra-vesical BCG or mitomycin), surgical approaches (e.g., radical cystectomy, nephroureterectomy), and systemic chemotherapy.

Oncologic outcomes included overall survival (OS), defined as the time from UC diagnosis to death or last follow-up; metastasis-free survival (MFS), defined as the time from UC diagnosis to the development of regional or distant metastases. Tumor recurrence was not captured in a standardized manner across the study period and was therefore not analyzed as a predefined endpoint in this cohort. For deceased patients, the cause of death was classified as UC-related, other malignancy, infection, or unknown. Imaging during post-transplant and oncologic follow-up was performed according to clinical practice and included ultrasonography, as well as cross-sectional imaging of the urinary tract (CT urography or MR urography) when clinically indicated.

### Statistical analysis

The incidence of UC was expressed as a standardized incidence ratio (SIR), defined as the ratio of observed-to-expected cases. The expected number of UC cases was derived by applying a population-based incidence rate of 11.05 per 100,000 person-years from Germany (calendar years 2019–2020) as reported in Krebs in Deutschland für 2019/2020 (German Centre for Cancer Registry Data at the Robert Koch Institute / GEKID) [19] to the total person-time at risk in the transplant cohort. Person-time at risk was calculated from the date of KT to the earliest of UC diagnosis, death, loss to follow-up, or administrative censoring (May 2024). Expected case numbers were calculated as (reference rate / 100,000) × total person-years at risk. The primary endpoint for the SIR analysis was de novo UC diagnosed after transplantation, combining bladder and upper-tract locations (native and graft-involving). Since population reference rates pertain to urothelial malignancies, we additionally performed a sensitivity analysis excluding non-urothelial histology. A 95% confidence interval (CI) for the SIR was estimated assuming a Poisson distribution.

Descriptive statistics were used to summarize demographic, transplant, and tumor-related characteristics. Categorical variables are presented as absolute numbers and percentages; continuous variables are reported as medians and ranges. Kaplan–Meier survival analysis was performed to estimate OS, MFS, and GS for descriptive purposes. Patients were censored at the time of last follow-up (‘last seen alive’) if the respective endpoint had not occurred. For MFS, death without documented metastasis was censored at the time of death; for GS, death with a functioning graft was treated as a censoring event. We acknowledge that event-free survival estimates may be overestimated in the presence of competing mortality. Loss to follow-up was operationalized as the date of the last documented contact (‘last seen alive’).

Statistical analysis was performed using IBM SPSS Statistics version 29 (Armonk, NY, USA) and JMP Pro version 18 (SAS Institute Inc., Cary, NC, USA). A p value < 0.05 was considered statistically significant. Due to the limited sample size, no multivariable analysis or matched control group was included in this study.

This study was conducted in accordance with the Declaration of Helsinki and was approved by the institutional ethics committee of Charité – Universitätsmedizin Berlin (approval number: EA1/252/22). The requirement for written informed consent was waived due to the retrospective nature of the study.

## Results

### Incidence of urothelial carcinoma after kidney transplantation

Among 4,012 KTRs transplanted between January 2005 and May 2024, 19 patients developed de novo UC during follow-up. This corresponds to a cumulative incidence of 0.47% and a standardized incidence ratio (SIR) of 2.21 (95% CI: 1.33–3.44), indicating a more than twofold increased risk compared to the general population. The cohort accumulated 77,800 person-years at risk. Based on the population reference rate (11.05 per 100,000 person-years), the expected number of UC cases was 8.60. Excluding the single non-urothelial histology (squamous cell carcinoma), the observed-to-expected estimate remained similar (SIR = 2.09, 95% CI 1.24–3.31).

### Baseline patient and transplant characteristics

Baseline characteristics are summarized in Table 1. The median age at KT was 56 years (range: 46–77), and most patients were male (15/19, 78.9%). The median body mass index (BMI) at time of transplantation was 23.5 kg/m2 (range: 15.4–30.9).

**Table 1:** Graft specific patient characteristics: values are shown as median (range) or number (percentage of the group)

Characteristics	UC (*n* = 19)
Age at KT (years)	56 (46–77)
BMI at KT (kg/m^2^)	23.5 (15.4–30.9)
Gender	
Male	15 (78.9%)
Female	4 (21.1%)
Donor age	60.5 (37–84)
Donor gender	
Male	7 (36.8%)
Female	9 (47.4%)
Unknown	3 (15.8%)
Living donor	3 (15.8%)
Location of renal transplant	
Left iliac fossa	10 (52.6%)
Right iliac fossa	9 (47.4%)
Underlying disease for CKD	
Diabetic nephropathy	4 (21.1%)
Nephrosclerosis	2 (10.5%)
IgA nephropathy	2 (10.5%)
Alport syndrome	2 (10.5%)
FSGS	1 (5.3%)
ADPK	1 (5.3%)
Hypertensive kidney disease	1 (5.3%)
Renal atrophy	1 (5.3%)
Tubulointerstitial nephritis	1 (5.3%)
Other	5 (31.6%)
Immunosuppression	
Tacrolimus	15 (78.9%)
MMF	15 (78.9%)
Ciclosporin	2 (10.5%)
Waiting time for KT (months)	36 (3–114)
Time of dialysis (months)	75.5 (7–108)
Remaining diuresis (ml)	400 (0–2750)
Type of dialysis	
Preemptive	1 (5.2%)
Hemodialysis	16 (84.2%)
Peritoneal dialysis	2 (10.5%)
CIT (min)	483 (164–850)
Operative time (min)	163.5 (89–300)
Follow up after KT (months)	108 (3–161)

A deceased donor was used in 84.2% of cases, while three patients (15.8%) received a living donor graft. The most common causes of end-stage renal disease were diabetic nephropathy (21.1%) and IgA nephropathy (10.5%). Median dialysis duration prior to transplantation was 75.5 months (range: 7–108), and median cold ischemia time was 483 minutes (range: 164–850).

At the time of UC diagnosis, the most frequently used immunosuppressive regimen included tacrolimus and mycophenolate mofetil (78.9%), while cyclosporine was used in 10.5% of patients. The median follow-up time after transplantation was 108 months (range: 3–161).

### Graft function and transplant-related outcomes

Graft-related outcomes are shown in Table 2. Major postoperative complications (Clavien–Dindo grade ≥3) occurred in three patients (15.8%), and delayed graft function was documented in nine patients (47.4%).

**Table 2:** Graft specific outcomes; values are shown as median (range) or number (percentage of the group)

Characteristics	UC (*n* = 19)
Major complication (CDC ≥ 3, 30 d)	3 (15.8%)
Delayed graft function	9 (47.4%)
Rejection	3 (15.8%)
Creatinine level (mg/dl)	
Preoperative	6.5 (3.9–11.9)
6 months post-KT	1.8 (0.8–4.8)
1 year post-KT	1.6 (0.9–8.6)
3 years post-KT	1.6 (0.7–3.0)
5 years post-KT	1.7 (0.8–3.7)
Graft failure	9 (47.4%)
Graft survival since KT (months)	140 (CI 108.26–171.74)
Graft survival since tumor diagnosis	Median not reached
Cause for graft failure	
Acute Rejection	1 (5.3%)
Tumor	4 (21.1%)
Primary non-function	1 (5.3%)
Death with non-functioning graft	3 (15.8%)
Transplant nephrectomy	3 (15.8%)

Graft failure occurred in nine patients (47.4%), including four cases (21.1%) directly attributed to UC progression. The median graft survival (GS) from transplantation was 140 months (95% CI: 108–172). Graft survival following UC diagnosis was not reached during the observation period.

Among functioning grafts, median serum creatinine remained stable, with a value of 1.7 mg/dL at five years post-KT.

### Tumor characteristics

Tumor-specific findings are presented in Table 3. The median age at UC diagnosis was 61 years (range: 50–80), and the median latency between transplantation and UC diagnosis was 47 months (range: 1–149). Latency from KT to UC diagnosis stratified by tumor location and by pathological stage (surgical subset) is provided in Supplementary Table 1. The most common presenting symptom was macrohematuria (21.1%), although many tumors were detected incidentally.

**Table 3:** Tumor-specific patient characteristics: values are shown as median (range) or number (percentage of the group)

Characteristics	UC (n = 19)
Age at tumor diagnosis (years)	61 (50–80)
Time until tumor (months)	47 (1–149)
Initial symptom	
Macrohematuria	4 (21.1%)
Microhematuria	1 (5.3%)
Dysuria	1 (5.3%)
None	13 (68.4%)
Tumor location	
Bladder	12 (63.2%)
UTUC native kidney	1 (5.3%)
UTUC transplant kidney	3 (15.8%)
Bladder + UTUC native kidney	1 (5.3%)
Bladder + UTUC transplant kidney	1 (5.3%)
Histology	
UC	18 (94.7%)
Squamous cell carcinoma	1 (5.3%)
Initial pT-Stadium	
pTa	8 (42.1%)
pT1	5 (26.3%)
pT2	3 (15.8%)
Unknown	3 (15.8%)
Clinical N1 Status at diagnosis	1 (5.3%)
Clinical M1 Status at diagnosis	2 (10.5%)
Grading (WHO 1973)	
G1	1 (5.3%)
G2	9 (47.4%)
G3	7 (36.8%)
Unknown	2 (10.5%)
Grading (WHO 2016)	
Low grade	8 (42.1%)
High grade	8 (42.1%)
Unknown	3 (15.8%)
Number of TURBTs	
0	1 (5.3%)
1	10 (52.6%)
2	4 (21.1%)
≥ 3	2 (10.5%)
Unknown	2 (10.5%)
Further therapy regime	
Radical cystectomy	3 (15.8%)
Partial cystectomy	1 (5.3%)
Nephroureterectomy	6 (31.6%)
BCG-instillation	1 (5.3%)
Mitomycin instillation	1 (5.3%)
Palliative chemotherapy (1st line Gemcitabine + Carboplatin or Cisplatin)	3 (15.8%)
Pathological T Stage of (partial) cystectomy or nephroureterectomy	(*n* = 10)
pTa	2 (20%)
pT1	2 (20%)
pT3	6 (60%)
Pathological positive lymph nodes	(*n* = 10): 1 (10%)
Death	10 (52.6%)
OS since KT (months)	108 (3–161)

UC was confined to the bladder in 63.2% of cases (12/19), while UTUC was diagnosed in six patients (31.6%), including three involving the transplant kidney. One patient had synchronous involvement of both the bladder and the graft.

Histologically, 94.7% of tumors were conventional UC, and one patient had squamous cell carcinoma.

Regarding tumor staging, the initial pathological T stage was pTa in eight patients (42.1%), pT1 in five (26.3%), and ≥pT2 in three (15.8%). One patient (5.3%) had clinically positive lymph nodes, and another presented with distant metastases. Tumor grading according to WHO 1973 classified 47.4% as G2 and 36.8% as G3. According to WHO 2016, 42.1% of tumors were low grade and 42.1% high grade. The number of TURBTs ranged from 0 to ≥3, with 52.6% of patients undergoing only one TURBT prior to definitive therapy.

### Treatment and oncological outcomes

Treatment modalities and outcomes are summarized in Table 4. Definitive surgical therapy included nephroureterectomy in six patients (31.6%), radical cystectomy in three (15.8%), and partial cystectomy in one patient. Among patients with transplant urinary tract involvement, definitive surgical management comprised nephroureterectomy of the transplant unit in three cases and ureteral reconstruction/re-implantation in one case in the context of combined bladder and transplant involvement. An overview of tumor location distribution and corresponding definitive treatment pathways is provided in Supplementary Figure 1. No relevant perioperative surgical complications were documented. Notably, graft failure with return to dialysis occurred in three of four transplant-involving cases following surgery. Systemic chemotherapy was administered to three patients (15.8%), and intra-vesical therapy (BCG or mitomycin) was used in two cases. One patient received no specific treatment due to severe comorbidities.

**Table 4:** Oncologic outcomes: values are shown as median (range) or number (percentage of the group)

Characteristics	UC (*n* = 19)
Synchronous metastases at diagnosis	2 (10.5%)
Metachronous metastases during follow-up	4 (23.5%)^†^
Metastasis-free survival	Median not reached
3-year MFS	73%
5-year MFS	73%
Death	10 (52.6%)
OS since tumor (months)	55 (CI 16.72–93.28)
3-year OS	70%
5-year OS	51%
Cause of death	*n* = 10
UC	3 (30%)
Other malignancy	2 (20%)
Pneumonia	1 (10%)
Sepsis	1 (10%)
Unknown	3 (30%)

^†^two synchronous metastatic cases were excluded; *n* = 17

**Fig. 1:**
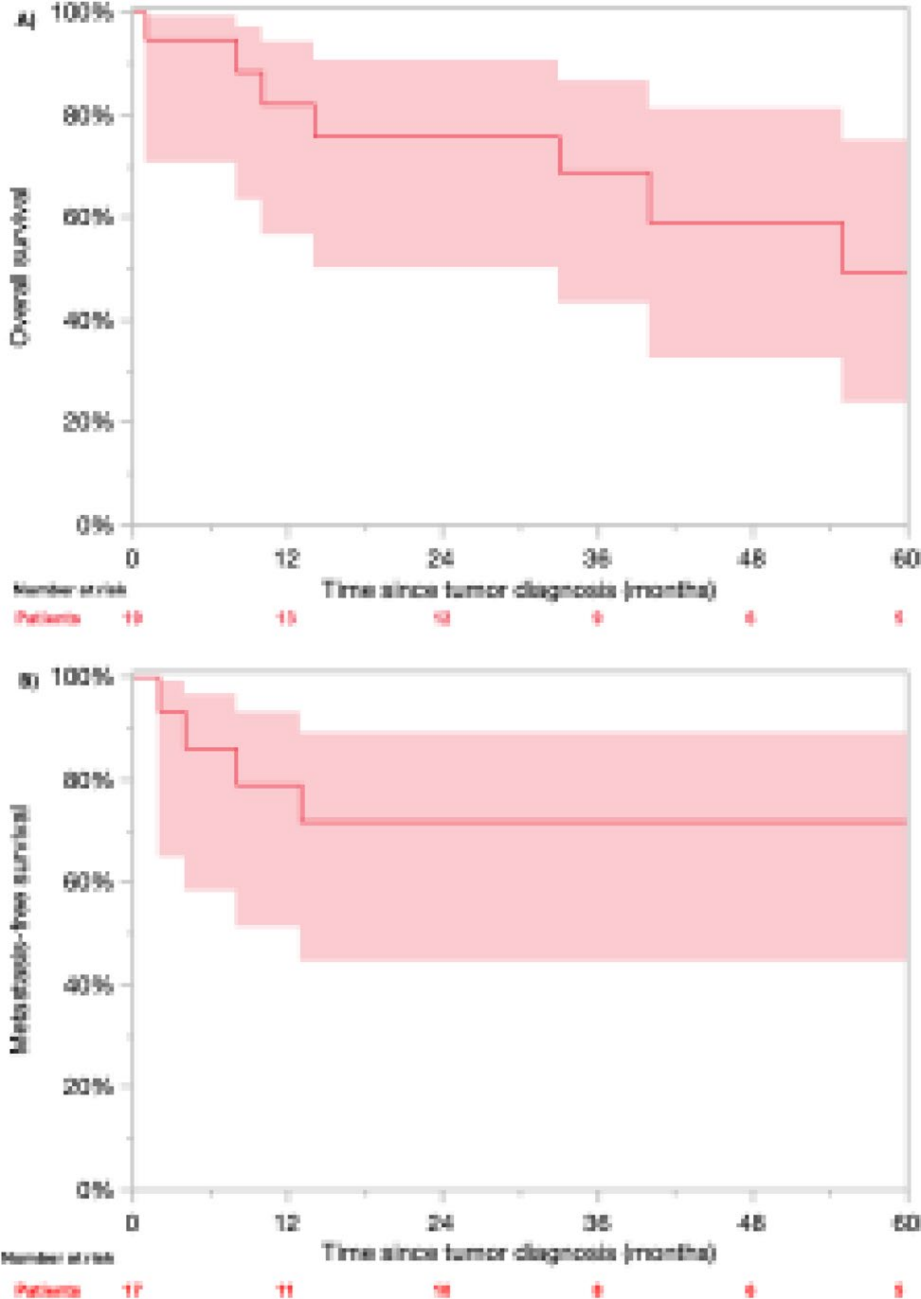
Overall and metastasis-free survival after UC diagnosis in kidney transplant recipients. Kaplan–Meier curves for **A** overall survival (event = death from any cause; patients alive at last follow-up were censored) and **B** metastasis-free survival (event = first regional or distant metastasis; patients without metastasis were censored at last follow-up or death without documented metastasis). Numbers at risk are shown below the curves

Kaplan–Meier survival analysis is shown in Fig. 1A (OS) and Fig. 1B (MFS).

At the time of last follow-up, 10 patients (52.6%) had died. The median overall survival (OS) after UC diagnosis was 55 months (95% CI: 16.7–93.3). The estimated 3-year OS rate was 70%, and the 5-year OS rate was 51%. UC was the primary cause of death in three patients (15.8%), while four deaths were attributed to other malignancies or infections. In three cases, the cause of death remained unknown.

Metastases developed in four patients (21.1%), primarily affecting lymph nodes and bone. The 3- and 5-year metastasis-free survival (MFS) rates were both 73%. Median MFS was not reached during the observation period.

**Fig. 2:**
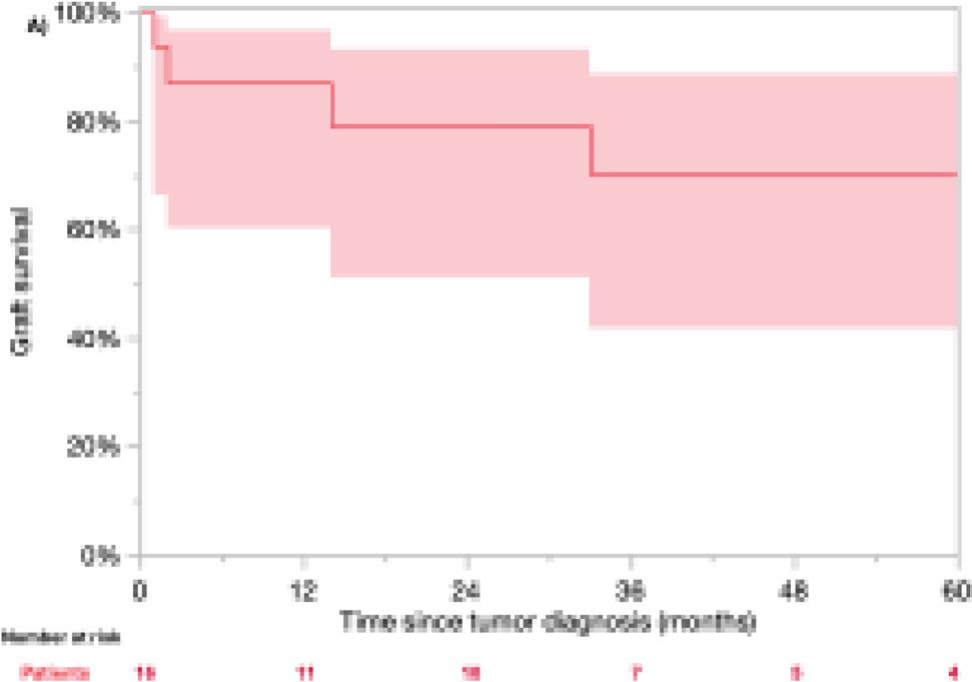
Graft survival after UC diagnosis. Kaplan–Meier curve of graft survival among recipients with a functioning graft at UC diagnosis (event = graft failure defined as return to dialysis, transplant nephrectomy, or death with a non-functioning graft; patients who died with a functioning graft were censored). Numbers at risk are shown below the curve

Graft function deteriorated in nine patients (47.4%), including three patients who underwent transplant nephrectomy as shown in Fig. 2. Four cases of graft failure were directly related to UC progression, and three patients died with a functioning graft.

## Discussion

This study provides a granular single-center real-world analysis of de novo urothelial carcinoma (UC) in kidney transplant recipients (KTRs), focusing on incidence, clinical presentation, treatment patterns, and both oncologic and graft-related outcomes. Despite being less common than renal cell carcinoma (RCC) in transplant cohorts, UC remains a clinically relevant malignancy, with diagnostic and therapeutic challenges due to the complex interplay of an aggressive entity, immunosuppression, chronic kidney disease (CKD), and the presence of a transplant.

In our cohort of 4,012 KTRs, 19 developed UC during follow-up, corresponding to a standardized incidence ratio (SIR) of 2.21, which indicates a moderately increased risk compared with the general population and aligns with registry-based analysis and meta-analysis reporting SIRs between 1.9 and 3.6 for UC after solid organ transplantation [1, 2, 20, 21]. For context, the SIR was lower than in our previous renal cell carcinoma (RCC) transplant cohort, but the present work focused on UC-specific patterns, particularly upper-tract and graft-involving disease [3].

A striking finding is the high proportion of upper-tract urothelial carcinoma (UTUC) in this cohort (31.6%), including three tumors involving the renal allograft, whereas UTUC represents only a small fraction of UC in the general population [4]. In contrast to several East Asian series reporting a female predominance and an even higher relative burden of UTUC and graft-involving tumors, our cohort was predominantly male, suggesting that regional and environmental exposures, such as aristolochic acid, may modulate disease patterns [4, 7]. Mechanistically, this distribution likely reflects a combination of transplant-related and regional risk factors, including aristolochic acid exposure, BK polyomavirus reactivation, ischemic injury, and long-term immunosuppression [4, 7, 10, 11].

Tumors arising in the transplanted kidney or ureter present clinical challenges due to the potential need for transplant nephrectomy, which directly jeopardizes graft function. In our cohort, nearly half of the patients experienced graft failure, and four cases were directly attributed to UC progression. These numbers reinforce findings from multi-center registries reporting graft-involving UC as a rare but impactful event [5, 14].

The median latency between transplantation and UC diagnosis was 47 months, which is somewhat shorter than previous reports of median latencies of 7–10 years [1]. However, the wide range (1–149 months) observed in our cohort highlights the need for lifelong surveillance. Several tumors in our series were detected incidentally, emphasizing that reliance on symptom-driven evaluation (e.g., macrohematuria) may result in delayed diagnosis.

Histologically, conventional UC dominated, and most patients had non–muscle-invasive disease (pTa or pT1). Still, 15.8% had muscle-invasive tumors (≥pT2), and 10.5% had distant metastases at diagnosis. Four patients (23.5%) developed metachronous metastases. While direct comparisons to immunocompetent populations are limited, our findings support prior reports suggesting a more aggressive disease course in immunosuppressed patients, potentially due to impaired immune surveillance, limited treatment options, and diagnostic delays [3, 6].

Therapeutic decision-making in this population remains complex. Several patients underwent radical surgery, including nephroureterectomy and cystectomy, and transplant nephrectomy was required in three patients—two for oncologic control and one due to graft failure. The need to balance oncologic efficacy against the risk of losing graft function is a core dilemma in this setting, particularly when the graft is directly involved.

Only two patients received intra-vesical therapy (one with BCG, one with mitomycin). The low use of BCG reflects clinical concerns over systemic infection and reduced efficacy under immunosuppression. While some case series suggest BCG may be safe in selected KTRs [15, 16], this remains controversial and requires individualized risk–benefit assessment.

Systemic therapy options are also limited. Platinum-based chemotherapy, the standard of care for advanced UC, is associated with nephrotoxicity and myelosuppression. Immune checkpoint inhibitors (ICIs), now integral to the treatment of metastatic UC, are rarely used in KTRs due to the high risk of allograft rejection—reported in up to 50% of patients [12, 17]. In our series, only three patients received systemic chemotherapy, and none received immunotherapy. This therapeutic gap underscores the urgent need for alternative treatment strategies for transplant recipients with advanced UC.

Despite these limitations, overall survival (OS) outcomes were relatively favorable: the 3-year OS was 70%, and the 5-year OS was 51%. However, these are inferior to our previous RCC transplant cohort (5-year OS: 72%) [18], again highlighting the potentially more aggressive behavior of UC in the transplant setting. UC was the cause of death in three of ten deceased patients (30%), with the remainder attributed to other malignancies, infections, or unknown causes.

**Fig. 3:**
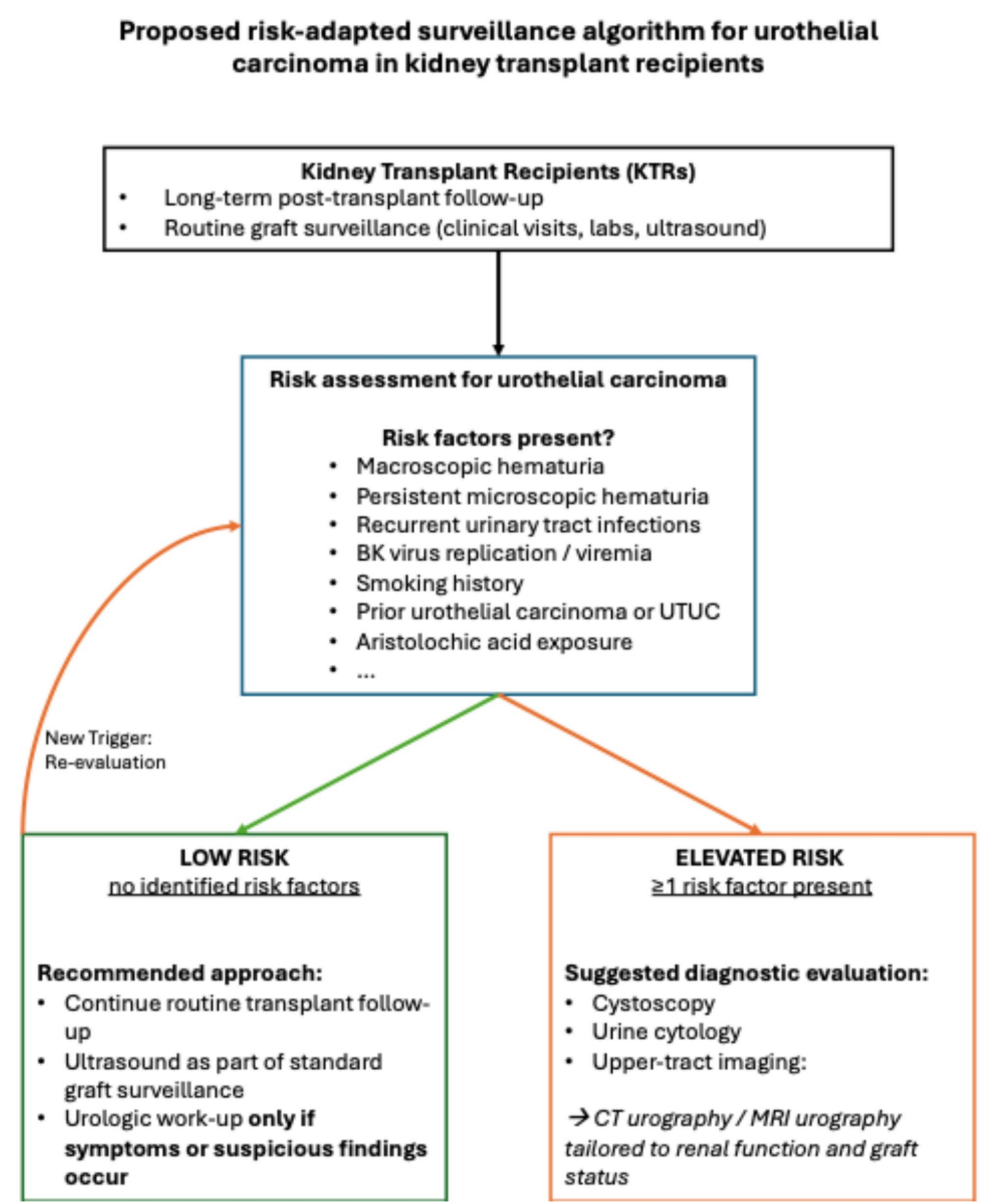
Proposed risk-adapted lifelong surveillance algorithm for urothelial carcinoma in kidney transplant recipients. The algorithm differentiates recipients without additional risk factors from those with ≥ 1 risk factor (e.g., smoking history, BK virus replication/viremia, suspected aristolochic acid exposure, prior UTUC, persistent hematuria) and outlines suggested triggers and diagnostic modalities (cystoscopy, cytology, and risk-adapted upper-tract imaging with escalation to CTU/MRU when clinically indicated)

From a diagnostic standpoint, our data—together with prior reports describing diagnoses well beyond a decade post-transplant [12, 13]—underscore the importance of tailored, risk-adapted surveillance protocols in KTRs. At our center, KTRs undergo structured post-transplant follow-up primarily focused on graft function, with regular clinical visits, laboratory monitoring, and ultrasound, whereas urologic evaluation is usually triggered by symptoms such as hematuria, recurrent urinary tract infections, unexplained graft dysfunction, or suspicious imaging findings. Annual ultrasound alone is unlikely to reliably detect early UC, particularly in the upper tract or transplanted kidney, and the wide latency range combined with a substantial burden of upper-tract and graft-involving tumors supports a risk-adapted lifelong surveillance approach Fig. 3. A low threshold for evaluating macroscopic or persistent microscopic hematuria with cystoscopy and upper-tract imaging appears warranted, and in patients with elevated risk—such as recurrent or persistent hematuria, recurrent infections, BK virus replication, smoking history, prior urothelial lesions, or suspected aristolochic acid exposure—intensified surveillance with periodic urine cytology and cross-sectional upper-tract imaging may be considered in addition to routine ultrasound-based follow-up.

This study has several limitations. First, the cohort is small because UC in KTRs is rare, which precluded multivariable analysis and age- or sex-stratified SIR estimation and necessitated an observed-to-expected approach using national reference rates. Second, the retrospective design relies on routine documentation; events occurring entirely outside our health system may have been missed, detailed information on aristolochic acid exposure, viral reactivation, and the exact anatomic sublocalization of graft-involving tumors was incomplete, and recurrence as well as cause-of-death attribution were not captured in a standardized manner across the full study period. Third, graft survival and metastasis-free survival were estimated using Kaplan–Meier methods without formal competing-risk modeling, which may overestimate event-free survival in a population with substantial competing mortality and should be refined in larger multi-center cohorts. Nevertheless, the structured transplant follow-up, high completeness of core data, and parallel reporting of oncologic and graft outcomes represent important strengths.

## Conclusions

Urothelial carcinoma remains a rare but clinically important malignancy in kidney transplant recipients, with a more than twofold increased incidence compared with the general population. This cohort demonstrates a disproportionate prevalence of upper-tract tumors, including graft-involving disease, and illustrates the diagnostic and therapeutic complexity of UC in the setting of chronic immunosuppression. Although overall survival was acceptable, graft loss occurred frequently and was particularly common in upper-tract and graft-involving tumors requiring radical surgery, underlining the tension between oncologic efficacy and preservation of transplant function. Immunosuppressive therapy restricts the safe and effective use of standard intra-vesical and systemic treatments, emphasizing the need for individualized, multidisciplinary management and for dedicated therapeutic strategies in this population. Given the broad latency window and potential for atypical presentations, lifelong urologic surveillance should be considered for KTRs, especially those with risk factors, such as BK virus infection, suspected aristolochic acid exposure, or persistent hematuria, and prospective multi-center studies are needed to refine risk-adapted surveillance intervals, imaging strategies, and systemic treatment options for immunosuppressed patients [12, 16].

## Supplementary Information

Below is the link to the electronic supplementary material.

Supplementary file1 (DOCX 38 KB)

## Data Availability

De-identified data underlying the main findings are available from the corresponding author on reasonable request, subject to institutional approvals and data-sharing agreements (GDPR compliance).

## Abbreviations

*ADPKD*: Autosomal dominant polycystic kidney disease
*BMI*: Body mass index
*BCG*: Bacillus Calmette–Guérin
*CDC*: Clavien–Dindo classification
*CI*: Confidence interval
*CIT*: Cold ischemia time
*CKD*: Chronic kidney disease
*DGF*: Delayed graft function
*ESRD*: End-stage renal disease
*GF*: Graft failure
*GS*: Graft survival
*KT*: Kidney transplantation
*KTR*: Kidney transplant recipient
*MMF*: Mycophenolate mofetil
*MFS*: Metastasis-free survival
*NMIBC*: Non-muscle-invasive bladder cancer
*OS*: Overall survival
*pT*: Pathologic tumor stage
*RCC*: Renal cell carcinoma
*SIR*: Standardized incidence ratio
*TURBT*: Transurethral resection of bladder tumor
*UC*: Urothelial carcinoma
*UTUC*: Upper-tract urothelial carcinoma
*WHO*: World health organization
