# Supplementary material for: De novo urothelial carcinoma in kidney transplant recipients: a single-center retrospective cohort study
Source: Int Urol Nephrol. 2026 Jan 21;58(7):2581–91. doi: 10.1007/s11255-026-05026-2 (PMC13309425; doi:10.1007/s11255-026-05026-2)
Supplement: Supplementary file 1 — Supplementary file1 (DOCX 38 KB) [file 11255_2026_5026_MOESM1_ESM.docx]

**Supplementary Material**

**Supplementary Table 1.** Time until tumor diagnosis after kidney transplantation stratified by tumor location and pathological T stage of cystectomy or nephroureterectomy (surgical subset); values are shown as median (range). Graft-involving disease corresponds to ‘UTUC transplant kidney’ and ‘bladder + UTUC transplant kidney’.

| **Characteristics** | **Time until tumor (months)** |
| --- | --- |
| Tumor location |  |
| Bladder (n=12) | 32 (1-149) |
| UTUC native kidney (n=1) | 99 |
| UTUC transplant kidney (n=3) | 47 (6-69) |
| Bladder + UTUC native kidney (n=1) | 71 |
| Bladder + UTUC transplant kidney (n=1) | 126 |
| Pathological T-Stage of cystectomy or nephroureterectomy |  |
| pTa (n=2) | 30 (13-47) |
| pT1 (n=2) | 70 (69-71) |
| pT3 (n=6) | 83 (6-126) |

**Supplementary Figure 1** Disease location distribution and management.
